# Climate and Dispersal: Black-Winged Stilts Disperse Further in Dry Springs

**DOI:** 10.1371/journal.pone.0000539

**Published:** 2007-06-20

**Authors:** Jordi Figuerola

**Affiliations:** Department of Wetland Ecology, Estación Biológica de Doñana, Consejo Superior de Investigaciones Científicas (CSIC), Seville, Spain; University of Edinburgh, United Kingdom

## Abstract

Climate affects the abundance and distribution of many species of wildlife. Nevertheless, the potential effects of climate on dispersive behaviour remain unstudied. Here, I combine data from (i) a long-term Black-winged Stilt (Himantopus himantopus) monitoring program, (ii) a capture-recapture marking program in Doñana, and (iii) reports from the Rare Birds Committee in the United Kingdom to analyse at different geographical scales the relationship between climate, survival, philopatry, and dispersive behaviour. Black-winged Stilt populations varied in size in consonance with changes in both the North Atlantic Oscillation (NAO) and local rainfall during the breeding season. Changes in population size are related to changes in philopatry and increases in dispersal beyond the traditional range of the species. The results indicate that climatic conditions influence the dispersive behaviour of individual birds, explaining rapid changes in the local population of this species breeding in unstable Mediterranean wetlands.

## Introduction

Climate affect the abundance and distribution of many species of wildlife. Impacts of last decades changes in climate include reduction in population size, survival, and/or productivity, and a northward shift in the distribution of a variety of different organisms [1]. North Atlantic Oscillation (NAO) index is one of the main cyclical climatic forces. During positive phases of the NAO, westerly winds increase temperature and rainfall over Northern Europe, and drought in the Mediterranean region [2]. Both ecological processes in the Sea (i.e. fish growth and survival) and the continent (i.e. competition between sympatric species, survival and productivity) seem affected by NAO phases [2]. Nevertheless, the potential effects of NAO and climate in general on dispersive behaviour remain unstudied. Theoretical studies predict major changes in the distribution of animals and plants in response to climate, although the validity of these models has yet to be established [3]. How do highly mobile organisms such as birds respond to changes in climate? I investigated the case of the Black-winged Stilt *Himantopus himantopus*, a common breeding wader in European and, in particular, in southern European wetlands [4]. The breeding population in Doñana (southwest Spain) is very variable, ranging from 50 pairs in dry years to over 14,000 pairs in wet years. Given that the total European population is estimated at 33,500–49,800 pairs, up to 28–42% of European Black-winged Stilts breed in some years in Doñana. Since 1988 the breeding population of Black-winged Stilts has been monitored by means of censuses of breeding pairs, the ringing of chicks with PVC rings, and the resighting of marked individuals.

## Results

**Figure 1:**
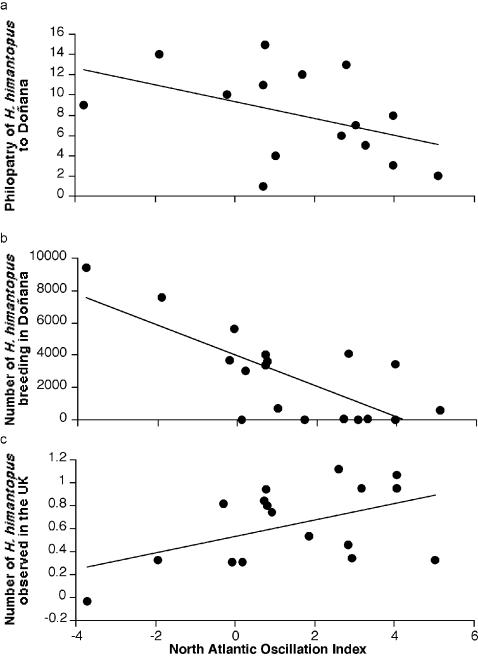
North Atlantic Oscillation and Black-winged Stilt population dynamics. a, Philopatry to breeding grounds estimated as resighting rates from the model Ø(age) p(time), *r* = 0.58, *F* = 6.49, *df* = 1, 13, *p* = 0.02, 1989–2003. b, Number of pairs breeding in Doñana National Park, *r* = 0.74, *F* = 19.78, *df* = 1, 16, *p* = 0.0004, 1988–2005. c, Frequency of Black-winged Stilt observations in the United Kingdom after controlling for the number of rare waders observed in the UK, a country outside the actual range of this species, *r* = 0.48, *F* = 4.15, *df* = 1, 14, *p* = 0.06, 1977–2004.

**Figure 2:**
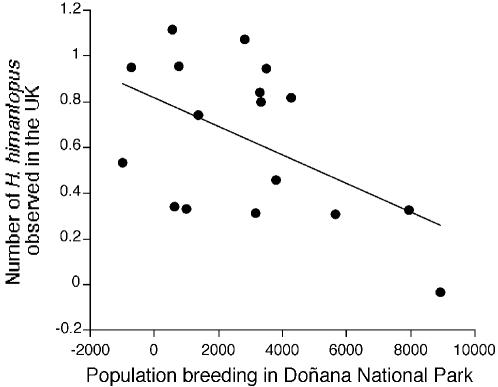
Population size in Doñana and Black-winged Stilt population dynamics. Frequency of Black-winged Stilt observations after controlling for the number of rare waders observed in the United Kingdom (*r* = 0.52, *F* = 5.00, *df* = 1, 14, *p* = 0.04).

I used capture-mark-resighting data spanning the years 1988–2003 to estimate Black-winged Stilt survival and resighting rates. Survival estimates are affected by mortality and permanent emigration, while resighting rates are affected by mark observation effort and temporal dispersal to other areas [5]. Survival is low during the first year of life (1^st^ year: 28.11%±12.75; 1+ year: 69.65%±4.78), although it was constant from one year to another. Resighting varied greatly with time and did not depend on age (Table 1). Time-dependent variation in resighting rates was better explained by local rainfall (45.1% variance explained) than by the NAO (33.5% variance explained, Figure 1a). The simultaneous combination of NAO and rainfall did not improve the fit on models including only rainfall. Breeding population size increased with resighting rates (*r* = 0.56, *F* = 5.95, *df* = 1, 13, *p* = 0.03) and rainfall during the breeding season (*r* = 0.64, *F* = 10.82, *df* = 1, 16, *p* = 0.005), but decreased with NAO (Figure 1b). Combining simultaneously NAO and rainfall increased the fit of the model (*r* = 0.86, *F* = 20.47, *df* = 2, 15, *p*<0.0001) and both factors remained significantly related to population size in Doñana (NAO, *F* = 18.37, *df* = 1, 15, *p* = 0.0006; rainfall, *F* = 10.01, *df* = 1, 15, *p* = 0.006). NAO (but not local rainfall in Doñana, *r* = −0.12, *F* = 0.22, *df* = 1, 14, *p* = 0.65) tended to be positively related, and Doñana population size negatively related to the frequency of observations of dispersing Black-winged Stilts in the United Kingdom, a country outside the range of the species (Figures. 1c and 2).

**Table 1:** Modelling the survival and resighting rates of Black-winged Stilts during the period 1988–2003 using capture-resighting data gathered in Doñana (southwest Spain).

a) Testing for age and time variation in survival and recapture parameters			
Model	QAICc	Parameters	QDeviance
Ø(age) p(time)	949.370	17	75.323
Ø(constant) p(time)	950.148	16	78.124
Ø(age) p(constant)	953.888	3	108.038
Ø(constant) p(constant)	954.072	2	110.226
Ø(age) p(age)	955.872	4	108.017
Ø(constant) p(age)	956.042	3	110.192
Ø(time) p(constant)	956.700	16	84.676
Ø(time) p(age)	958.550	17	84.503
Ø(age) p(age*time)	963.328	31	60.818
Ø(time) p(time)	968.177	29	69.750
Ø(age*time) p(constant)	968.765	30	68.297
Ø(age*time) p(age)	970.756	31	68.247
Ø(time) p(age*time)	983.817	44	54.637
Ø(age*time) p(time)	985.683	44	56.503
Ø(age*time) p(age*time)	1000.515	58	42.349
Ø(constant) p(age*time)	1036.888	30	136.421
b) Testing for the effects of weather on model parameters in the model Ø(age) p(time)			
Ø(age) p(rain March–August)	941.131	4	93.275
Ø(age) p(NAO+rain March–August)	941.183	5	91.321
Ø(age) p(NAO)	944.958	4	97.103
Ø(age) p(time)	949.370	17	75.323
c) Testing for effects of weather on model parameters in the model Ø(constant) p(time)			
Ø(constant) p(rain March–August)	941.497	3	95.647
Øconstant) p(NAO+rain March–August)	941.472	4	93.617
Ø(constant) p(NAO)	944.997	3	99.147
Ø(constant) p(time)	950.148	16	78.124

Models are defined in terms of survival (Ø) and recapture rates (p) that vary between first-year and adult birds (age) and between years (time). AIC was adjusted for a c-hat value of 1.12. The models supported by the AIC criterion during the model selection process are marked in bold.

## Discussion

Considerable attention has been focused on the incidence of climatic factors on survival, reproduction, and/or phenology [6], [7]. However, the impact of climate on philopatry and dispersal has received much less attention [8], [9]. The results of this study suggest that climatic conditions have important effects on birds' philopatry, which is in turn related to changes in breeding populations. Ultimately these changes lead to an increase in dispersive behaviour and an improved capacity for colonizing new breeding localities. NAO, one of the Earth's main large-scale cyclical climatic patterns, is negatively associated to rainfall in Spain and North Africa [10]. Both local rainfall and NAO have been related by multivariate analyses to breeding population size in Doñana: the former is a better estimator of habitat availability for birds migrating into Doñana, while the latter may affect the probability that birds wintering in Africa will breed closer to their wintering grounds. Unfortunately, no information is available on numbers of Black-winged Stilts breeding in North African countries to directly test this relation between NAO and African breeding distribution. The resighting rate is not only affected by philopatry, but also by other factors such as research effort and consequently analyses of this factor should be considered conservative (and may explain the moderate amount of variance explained by the models). Despite this, the resighting rate is clearly associated with population changes in the study area and in a region more than 1500 kilometres from Doñana, a fact that increases confidence in the soundness of results.

Temperature increases leading to rising sea-levels [11] and changes in rainfall patterns [12] will harm coastal wetlands. In addition to cyclical fluctuations, a long term trend towards more positive NAO is occurring [2]. While rain will probably increase in northern Europe, the Mediterranean region will become drier [12] and under this scenario information on how weather influences the demographic parameters of organisms is urgently needed. In terms of survival I found no direct costs of dispersal, although the effects on individuals fitness cannot be excluded given that other studies have found a relationship between dispersal and delayed breeding [13], [9].

Understanding how species' ranges are modified in light of climate change is an important issue in ecology. Travis & Dytham [14] have recently produced a model that suggests that during range expansion selection may favour individuals with a higher propensity for dispersal. A paradoxical result of my study is that climate itself can modify the propensity of individuals to disperse, thereby increasing the probability of range expansion in species that live, for example, in unstable environments such as Mediterranean wetlands.

## Materials and Methods

### The data

Since 1988 the number of breeding pairs of Black-winged Stilts has been counted every year in the Doñana National Park and the resulting figures have been found to be closely correlated to the numbers counted in the whole of Doñana (*r* = 0.93, *F* = 69.03, *df* = 1, 10, *p*<0.0001), which are available for far fewer years.

The observation of rare birds outside their normal ranges has long been a common hobby in many countries and the British Birds Rarities Committee revised and evaluated all observations of rare birds in Great Britain since 1959. This task provides valuable information regarding the frequency of the rare bird species that wander to Great Britain and can be consulted on the Internet (http://www.bbrc.org.uk/waders.htm). Observations from all years between 1988 and 2004 were included in the analyses. The number of observations in any given year or territory will clearly depend on the number of ornithologists living or visiting that area. To control for differences in observer effort, the number of any other rare species of waders (Infraorder Charadriides, excluding the family Laridae; 15) observed in the UK was included as a covariate in the analyses. Both the numbers of Black-winged Stilts and waders observed in UK were log transformed.

Two different indicators of environmental conditions were used in the analyses: rainfall in Doñana from March to August (the Black-winged Stilt breeding season) (available at http://www-rbd.ebd.csic.es/Seguimiento/mediofisico/parametrosmeteorologicos/em05.htm), and the mean winter NAO-index (available at http://www.cgd.ucar.edu/cas/jhurrell/indices.html). NAO is a major source of interannual variability in atmospheric circulation and the NAO index is estimated on the basis of differences in pressure at sea-level between Lisbon and Reykjavik from December to March.

### Modelling survival and resighting rates

Chicks have been ringed in Doñana since 1988 with metal rings and white plastic DARVIC rings marked with a black, three-digit code. These rings can be read from a distance with the aid of a telescope. I conducted a capture-resighting analysis to estimate survival and resighting rate using the program MARK and information pertaining to 2964 individuals ringed and observed in Doñana during the period 1988–2003. Only captures and observations occurring between March and August were included in the analyses so as to estimate the probability of a bird alive in one breeding season being still alive during the next breeding season.

Survival analysis was based on the Cormack-Jolly-Seber models (CJS). Survival rates are affected both by mortality and by permanent emigration from the study area and resighting rates reflect both variation in observation/capture effort and the temporary emigration of individuals from the study area [16]. The relationship between climatic variables and survival and/or recapture parameters was tested using a capture-recapture model selection and simplification approach [5]. Model selection was based upon a fully parameterized model in which both survival and recapture probabilities varied with age and time. Models incorporating age-dependent effects only allowed parameters to be different from adults during the year after capture as chicks. The adjustment of the CJS model to the data was assessed with the RELEASE programme's goodness-of-fit test [17] and with a parametric bootstrap approach. The parameteric estimates from the model were used to simulate data in line with the assumptions contained in the CJS models (individuals were independent and no overdispersion of data occurred). This process was repeated 1000 times and the deviance of each model was calculated to determine whether the deviance of the observed model exceeded that of the simulated data. The overdispersion parameter (c-hat) was calculated as the ratio between the mean deviance of simulated models and the deviance of the observed model [18]. We found no evidence of significant overdispersion (c-hat = 1.12) and thus no evidence for the assumption that fates of individual birds were independent of each other [19]. We adjusted results to a c-hat of 1.12 (ideally c-hat should be 1.00), although this adjustment had no qualitative effect on the results.

Model simplification was based on an analysis of the factors affecting survival and resighting probabilities and constructed models with variation between age and time, and the age*time interaction. Finally, using the simplified capture and survival model, we tested the possible relationships between resighting rates, which were found to vary annually, local rainfall and NAO. I used a small sample size adjusted by Akaike's information criterion (AICc) for model selection. AICc is considered to be a simple, effective, and objective means for model selection [20]. Models with lower AICc values are assumed to best fit the data with the least possible number of parameters. Models with AICc values differing by less than 2 were considered to be equivalent. The amount of variance explained by climatic variables was calculated as Deviance(model constant) - Deviance(model with covariate) / Deviace(model constant) - Deviance(model with time dependent parameter), as per [21].

### Statistical analyses

A Pearson regression was used to analyse the relationship between NAO and rainfall in Doñana, and breeding population size and frequency of observations in the UK.
